# Exercise Reverses Dysregulation of T-Cell-Related Function in Blood Leukocytes of Patients With Parkinson's Disease

**DOI:** 10.3389/fneur.2019.01389

**Published:** 2020-01-28

**Authors:** Yong Hu, Kunshan Zhang, Tianyu Zhang, Junbang Wang, Fei Chen, Wenting Qin, Weifang Tong, Qiang Guan, Yijing He, Chunya Gu, Xiaoyu Chen, Un Jung Kang, Yi E. Sun, Siguang Li, Lingjing Jin

**Affiliations:** ^1^Department of Neurology, Shanghai Tongji Hospital, Tongji University School of Medicine, Shanghai, China; ^2^Department of Neurology, Department of Neuroscience and Physiology, NYU Langone Health, The Marlene and Paolo Fresco Institute for Parkinson's and Movement Disorders, Neuroscience Institute, New York, NY, United States; ^3^Stem Cell Translational Research Center, Tongji Hospital, Tongji University School of Medicine, Shanghai, China; ^4^Department of Spine Surgery, Shanghai Tongji Hospital, Tongji University School of Medicine, Shanghai, China

**Keywords:** exercise, rehabilitation, Parkinson's disease, gene expression profile, inflammatory cytokine, T cell

## Abstract

Parkinson's disease (PD) is a common neurodegenerative disease with movement and balance impairments. Although studies have reported improvement of motor symptoms with physical exercise, the mechanisms by which exercise is beneficial remains poorly understood. Our study addresses the exercise-induced changes to peripheral immune cells by interrogating the transcriptome of blood-derived leukocytes in PD patients before and after exercise. Patients attended 1 h exercise classes twice a week for 12 weeks. Leukocytes were collected at the beginning and end of the study for gene expression analysis by RNA-seq or quantitative real-time PCR. We correlated differentially expressed genes after exercise with clinical measures and analyzed the potential functions of gene changes with Kyoto Encyclopedia of Genes and Genomes pathway and Gene Ontology analysis. Exercise improved measures of movement and balance when compared with scores before the exercise program. Among the gene changes, Kyoto Encyclopedia of Genes and Genomes and Gene Ontology analysis suggests that T-cell receptor signaling, T-cell activation, and T-cell migration pathways were downregulated, while the T-cell receptor signaling pathway was the most significantly correlated with clinical measures. To further investigate T-cell-related changes in PD leukocytes, we reanalyzed the differentially expressed genes from publicly available microarray data and found that genes in the T-cell activation, differentiation, and migration pathways were upregulated in PD samples compared to controls in a time-dependent manner. Together, our findings suggest that exercise rehabilitation may improve movement and balance in PD patients by reversing the upregulated T-cell activation pathways associated with PD. This study was registered with the Chinese Clinical Trial Registry under ChiCTR-TRC-14004707. Registered on May 27, 2014.

## Introduction

Parkinson's disease (PD) is a common neurodegenerative disease characterized by loss of dopaminergic neurons in substantia nigra and accumulation of aggregated alpha-synuclein in the brain stem, spinal cord, and cortical regions (1). Movement and balance impairments, especially postural instability, adversely affect the daily function and quality of life of patients with PD (2). Although motor symptoms are alleviated with drug therapy, balance impairments are not optimally controlled by pharmacotherapy and require alternative approaches. Exercise has been shown to slow the deterioration of motor symptoms and prolong functional independence (2–4), improve movement (3, 4) and balance (5), and decrease the incidence of falls (5–9). As such, exercise has been an integral part of PD management, although the underlying mechanisms by which exercise confers benefit remain controversial. Studies have suggested that exercise improves neurological function through neuroprotection (10, 11), improved neurogenesis, increased neuroregeneration (12, 13), and cardiorespiratory rehabilitation (14), although these findings have not been fully replicated (15). Accordingly, the mechanisms by which exercise is beneficial in patients with PD have not been fully elucidated.

Many neuron-intrinsic factors likely contribute to PD pathogenesis, including mitochondrial dysfunction, increased oxidative stress, and proteasomal dysfunction. In addition, increasing evidence suggests that perturbation of the immune system may play an important role in PD pathogenesis (16–18). While central inflammation is widely recognized as a pathological hallmark of PD and may underlie neuronal death in the substantia nigra (19), mounting evidence suggests that peripheral inflammation contributes to the overall disease. In PD patients, blood–brain barrier impairment has been reported (20), and leukocytes have been identified near dopaminergic neurons in postmortem brain tissue from PD cases (21). In addition, many of the pathways implicated in neuronal dysfunction are also perturbed in peripheral blood cells from patients with PD, including altered ubiquitin–proteasome and mitochondrial pathways and the presence of alpha-synuclein peptides (22, 23). In addition, evidence has suggested that peripheral inflammation plays a role in the early stages of disease initiation and progression, including the development of preclinical non-motor symptoms (19). These findings are supported studies manipulating peripheral immune cells in mouse models of PD, whereby inhibiting immune cell infiltration can attenuate dopaminergic neuron loss (24, 25). Together, these studies suggest that peripheral immune responses may contribute to the pathogenesis of PD, and modulation of the immune system may be beneficial in the treatment of PD.

In healthy individuals, prolonged and regular physical exercise has been shown to promote an anti-inflammatory environment or attenuating the acute response to exercise (26, 27). Whether exercise has the same effect in PD patients has been largely unexplored, and the relationship between such effect and PD clinical symptoms remains unknown. To address these, we obtained gene expression profiles from peripheral leukocytes of PD patients before and after participating in an exercise program and correlated changes in gene expression profiles with clinical measures of movement and balance. In addition, we compared our experimental results with own analysis of a publicly available microarray dataset (GSE99039) and a microarray dataset from Parkinson's Progression Marker Initiative (PPMI).

## Materials and Methods

### Clinical Study

Patients with PD were recruited at the Shanghai Tongji Hospital by neurologists using the United Kingdom Parkinson's Disease Society Brain Bank clinical diagnostic criteria. Patients with confounding factors affecting the immune system were excluded. Two groups of patients on stable medication use were enrolled in this study, and they were asked to stay on the original medication dosage during the entire study. Blood samples of group 1 were used for RNA sequencing, while samples of group 2 were used to confirm the findings of group 1 with different methods (PCR and chemiluminescence immunoassay).

Participants were randomly allocated to tai chi (a kind of traditional Chinese martial arts) or multimodal exercise training (MET) group by researchers using a random numbers generator program, in a ratio of 1:1. The MET consisted of four exercise programs (core stability training, cycle ergometer, cross-obstacle training, and standing on ankle joint correcting board). Interventions were then performed as described before (28). The random number was delivered in a sealed envelope to each enrolled patient. Both groups attended exercise classes twice a week for 12 weeks. Assessments were completed by clinicians who were blinded to the mode of exercise intervention before and at the end of the 12th weeks.

The effect of exercise on PD was assessed by Parkinson's Disease Rating Scale Motor Examination (UPDRSIII), stride length, gait speed, Timed Up and Go Test (TUG), and the Berg Balance Scale (BBS). Movement was assessed by UPDRSIII (29), stride length, gait speed, and TUG (30). Stride length and gait speed were assessed with a 10 m walk. BBS (31) was used to measure balance. Among these clinical measures, a higher score of UPDRSIII and TUG demonstrates worse symptoms that resulted in a lower score in stride length, gait speed, and BBS. The human experiment was approved by the Ethics Committee of Tongji Hospital, performed in accordance with the Declaration of Helsinki, and was registered with the Chinese Clinical Trial Registry under ChiCTR-TRC-14004707. Written informed consent was obtained from each participant.

### Blood Sample and RNA Isolation

Peripheral venous blood samples in fasting state were collected by a standard procedure for complete blood counts and RNA isolation before the start and 48–72 h after the completion of the 12 weeks training program. Complete blood counts for white blood cell analysis were obtained from the clinical hematology laboratory by standard methods. Ethylenediaminetetraacetic acid was used as the anticoagulant for the isolation of peripheral blood leukocytes. The leukocytes were isolated and stored in TRIzol Reagent (Life Technologies) at −80°C until RNA isolation was completed. Total RNA was extracted using TRIzol Reagent (Life Technologies) following the manufacturer's protocol for whole blood. RNA concentrations were assessed by running a small amount of each sample on Nanodrop 2000/2000C (Thermo Fisher Scientific). The RNA quality was checked using the Agilent 2100 Bioanalyzer.

### RNA Sequencing

Total RNA was depleted of globin messenger RNA using the GLOBINclear-Human Kit (Life Technologies). Whole transcriptome libraries were made as outlined in the Ion Total RNA-Seq Kit v2 (Life Technologies). Selected libraries were diluted according to the final concentration of 10 pM and clonally amplified using the Ion PI Template OT2 200 Kit v3 (Life Technologies). These amplified libraries were then purified and sequenced on the Ion Proton sequencer for a total of six sequencing runs (Ion PI Chip kit v2) (Life Technologies) according to the manufacturer's instructions.

### Transcriptome Analysis

All reads were mapped to the human genome (hg19) by Tophat2 (v2.01). Unmapped reads were mapped again with bowtie2 (v2.10) in local mode. Alignment results were merged by the SAMtools. Normalized fragments per kilobase of transcript per million fragments mapped of genes in the RefGene annotation (2015 Feb) were estimated by Cuffquant (Cufflinks v2.21) with [–b, –u] options followed by Cuffnormal (Cufflinks v2.21). Samples in two conditions (before and after exercise) were applied to paired student *t*-test to avoid individual differences. Significantly differentially expressed genes (DEGs, *P* < 0.05) were used for enrichment analysis. Enrichment analysis including Gene Ontology (GO) analysis and Kyoto Encyclopedia of Genes and Genomes (KEGG) pathway analysis was performed by the Database for Annotation, Visualization, and Integrated Discovery. Paired centralized expression levels of each gene in each individual between two conditions were applied to principal component analysis. The microarray data of GSE99039 (22) was downloaded from the NCBI GEO database (http://www.ncbi.nlm.nih.gov/geo/) and analyzed by LIMMA. The microarray data of PD at baseline and 6 months after baseline was downloaded from the PPMI database (www.ppmi-info.org/data).

### Real-Time qPCR

For confirmation of RNA-Seq findings, selected candidate genes were validated by quantitative real-time PCR (RT-qPCR). Total RNA was used to synthesize complementary DNA using Prinmascript RT Mastermix (Takara). All primers were designed using Primer3 (http://bioinfo.ut.ee/primer3/), synthesized by Sangon Biotech (see Table S1). BLAST searches were performed to confirm the specificity of the primer sequences. PCRs were carried out for 40 cycles (95°C for 15 s and 60°C for 60 s) using QuantStudio 7 Flex Real-Time PCR System (Applied Biosystems). The PCR reaction mixture (10 μl) contained 1 μl of complementary DNA, specific primer sets, and Power SYBR Green PCR Master Mix (Applied Biosystems). The relative levels of selected genes were normalized to glyceraldehyde 3-phosphate dehydrogenase.

### Cytokine Quantification in Plasma

Cytokine levels of interleukin (IL)-6, IL-2R, and tumor necrosis factor (TNF) in sera of group 2 were quantified by chemiluminescence immunoassay with IMMULITE 1000 Immunoassay System (SIEMENS) according to the instructions of the manufacturer. All samples were measured by the average of two replicates of each sample. Concentrations below the detection limit were considered undetectable.

### Statistical Analysis

Statistical analyses were carried out using SPSS (SPSS Inc., Chicago, Illinois, USA) version 20.0. Between-group differences in demographic and baseline variables were tested with non-parametric Mann–Whitney *U*-test and χ^2^-test for categorical variables and independent sample *t*-test for continuous variables. A normal distribution test was performed before each *t*-test that analyzed only normally distributed variables. Intervention effects on continuous outcome measures were compared by repeated-measures analysis of variance with two groups as the between-subjects factor and time points as the within-subjects factor. Significance differences between the groups were tested with an independent sample *t*-test. Paired *t*-tests were used to examine within-group changes from baseline to the completion point of the 12 weeks exercise program. The significance level was set at *p* < 0.05, and all tests for significance were two-tailed.

## Results

### Exercise Improved Movement and Balance in Patients With PD

From June 2014 to July 2015, 43 participants were consecutively randomized in group 1 (10 in the tai chi group and 11 in the MET group) and group 2 (11 in the tai chi group and 11 in the MET group) (Figure S1). Three of the 43 participants did not complete their assigned interventions (one in the tai chi group of group 2 and two in the MET group of group 2).

**Figure 1:**
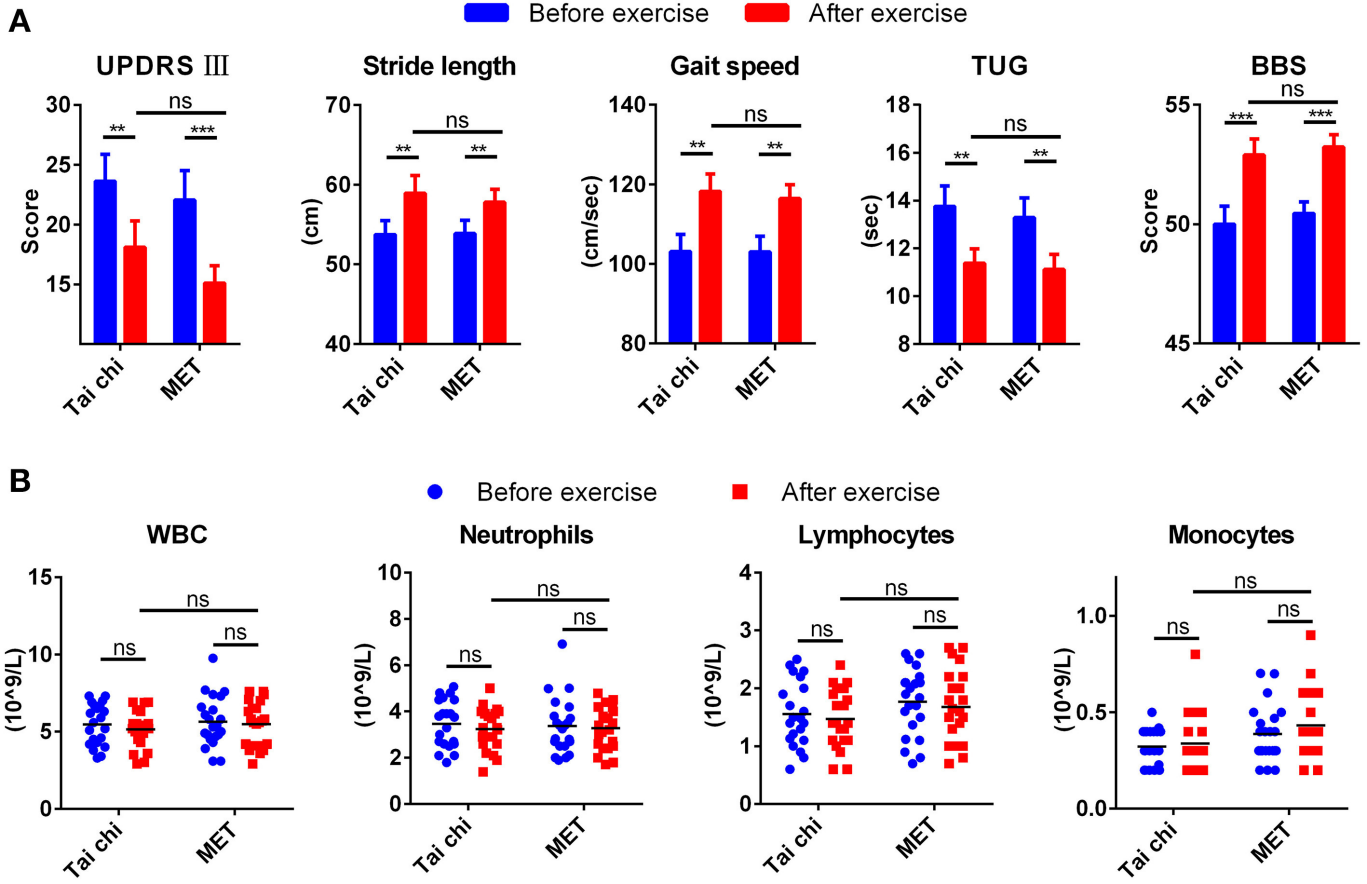
Clinical measures and leukocyte response to exercise. **(A)** Exercise improved clinical measures for movement and balance in patients with Parkinson's disease (PD). Data are shown in mean ± SEM. **(B)** Exercise did not change the count of leukocyte types in patients with PD. MET, multimodal exercise training. ^*^P < 0.05; ^**^P < 0.01; ^***^P < 0.001; ns, not significant.

Table S2 summarizes the demographic characteristics, and no significant difference was observed between tai chi and MET group in all demographic characteristics. After a 12 weeks exercise program, patients from both groups demonstrated better performance in movement and balance compared with baseline regarding stride length, gait speed, TUG, and BBS (Figure 1A). They even achieved a lower score for UPDRS III compared with baseline, from 23.62 ± 10.36 to 18.10 ± 10.05 in the tai chi group and from 22.05 ± 11.47 to 15.10 ± 6.78 in the MET group (Figure 1A). In addition, no significant difference was observed between the two groups (tai chi and MET) in the five outcome measures (Figure 1A).

### Count of Leukocyte Types in PD Patients Remained Similar After Exercise

Peripheral venous blood samples from patients with PD were collected before and after exercise to test the affection of exercise on the count of blood leukocytes types. The number of total white blood cells, neutrophils, and lymphocytes decreased mildly after exercise, while monocyte mildly increased. None of them experienced a significant change (Figure 1B).

### Exercise Regulated the Expression of Genes in Leukocytes of PD Patients

**Figure 2:**
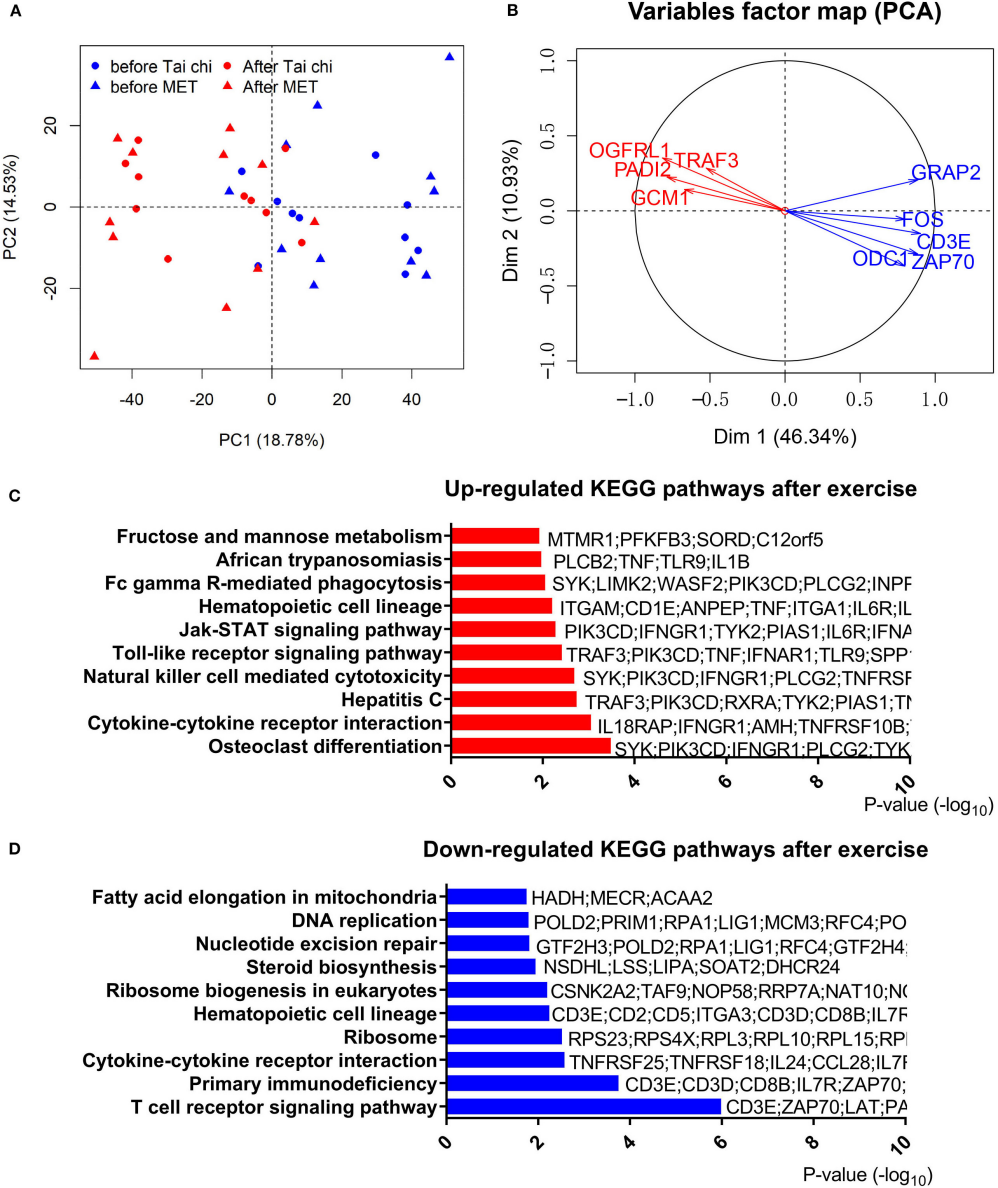
Analyses of differentially expressed genes in the leucocytes of Parkinson's disease (PD) patients before and after exercise. **(A)** Principal component analysis of differentially expressed genes after centralized distinguishes between before and after exercise. PC, principal component; MET, multimodal exercise training. **(B)** Distinct gene groups characterized after exercise and before exercise groups on the basis of differential correlation with Dim 1 and Dim 2. Arrow tip denotes correlation coefficient of the distinct gene with each Dim. Dim, dimension. **(C)** Top 10 upregulated Kyoto Encyclopedia of Genes and Genomes (KEGG) pathways after exercise. **(D)** Top 10 downregulated KEGG pathways after exercise.

To address the effect of exercise on leukocytes of PD patients, we performed RNA-Seq with leukocytes from 42 blood samples (21 PD patients of group 1 with every one taking a blood test before and after the exercise program), and the other 38 samples (19 PD patients of group 2) were used for RT-qPCR to confirm results of RNA-Seq. Principal component analysis of RNA-Seq data showed that samples in different conditions (before or after exercise) were separated (Figure 2A). However, consistent with clinical measures, the type of exercise (tai chi and MET) did not lead to a significant transcriptomic difference. Thus, in the following analysis, we combined the two groups (tai chi and MET) to explore the shared transcriptome differences driven by exercise. In the analysis of RNA-Seq data, 1,873 genes were identified as DEGs in the before- and after-exercise group (Figure 2B, Table S3). Among them, 1,453 DEGs had higher expression levels before exercise, while 417 DEGs had higher expression levels after exercise.

The most significantly downregulated gene after exercise was ODC1, which is functionally associated with PINK1, a causal gene responsible for hereditary recessive early-onset Parkinsonism (32). We also noted that CD3E, part of the TCR–CD3 complex on the T-lymphocyte cell surface, was notably downregulated after exercise. The CD3E complex mediates signal transduction, resulting in T-cell activation (33). TRAF3, which was upregulated after exercise, is an essential constituent of several E3–ubiquitin–protein ligase complexes and might promote ubiquitination of target proteins (34). The significant downregulation of ODC1, CD3E, and upregulation of TRAF3 after exercise were confirmed by RT-qPCR in a separate validation cohort (group 2) (Table S4). In addition, we found that the expression of SNCA, GBA, PARK, LRRK2, PRKN, and PINK1, which are common genetic causes for PD, were not regulated by exercise.

Abnormal aggregation of α-synuclein played a crucial role in the pathogenesis of synucleinopathies in PD (23, 35). In our previous study, AEP/PIKE-L axis was proved to regulate α-synuclein-related etiopathological effects (35, 36). Our RNA-Seq data showed that, after exercise, both LGMN and AGAP2 exhibited a pattern indicating the amelioration of PD pathogenesis (Figure S2), in which AGAP2 was significantly upregulated after exercise.

### T-Cell-Related Programs Were Downregulated After Exercise

To further understand the effect of physical exercise, we performed a functional analysis of DEGs. We observed 22 upregulated terms and 14 downregulated terms by KEGG pathway analysis (Figures 2C,D, Table S5). T-cell receptor signaling pathway and primary immunodeficiency pathway were highly enriched in downregulated genes, while the osteoclast differentiation pathway was the most enriched in upregulated genes after exercise. Consistent with the RNA-Seq results from group 1, members of the T-cell receptor signaling pathway (CD3E, GRAP2, FOS, and ZAP70) were significantly downregulated, which was confirmed by qPCR in group 2 (Table S4). These results indicated that the change in T-cell-related functions could be regulated by exercise.

Notably, the cytokine–cytokine receptor interaction pathway was enriched in both modulations (up- and downregulated) with different genes (Figures 2C,D, Table S5). In these terms, we observed an upregulation of TNF and a downregulation of IL-2RA/B after exercise. To confirm the expression pattern of TNF and IL-2RA/B, we analyzed TNF and IL-2R levels in the serum of a different group of patients with PD (group 2, Figure S3). Similar to RNA-Seq results, an upregulation of proinflammatory TNF and a tendency for downregulation of IL-2R after exercise were observed in group 2. However, IL-6, a key inflammatory factor that was elevated in the serum of PD patients, showed no significant changes in the plasma protein and transcript levels. Furthermore, inflammation response transcription factor CEBPB (Figure S2) showed a downregulated trend after exercise, consistent with the downregulation of many inflammatory cytokines.

GO enrichment analysis highlighted the inflammation and T-cell-related pathways as well. We observed that “positive regulation of response to external stimulus” was enriched in the upregulated genes after exercise (Table S6). In addition, for downregulated genes after exercise, the most significantly enriched terms were genes involved in positive regulation of leukocyte proliferation and transferase activity (Table S6). The downregulation of “positive regulation of leukocyte proliferation,” which included T-cell activation regulating genes (CCL5, CD28, CD3E, IL23A, IL2RA, PRKCQ, TMIGD2, TNFSF9, ZAP70, and so on), was consistent with the dysfunction of T-cell receptor signaling pathway in KEGG pathway analysis. We observed that the term of SUMO ligase activity was enriched in the upregulated genes after exercise (Table S6), which included genes regulating the ubiquitin–proteasome system. We also found that genes related to mitochondrion organization, mitochondrial membrane, mitochondrial inner membrane, and mitochondrial fission were regulated after exercise (Table S6), but they were not among the most significantly regulated terms, indicating that the mitochondrion function was not tremendously affected by exercise.

### Changes in the Genes for T-Cell Activation Correlated With Movement and Balance in PD

**Figure 3:**
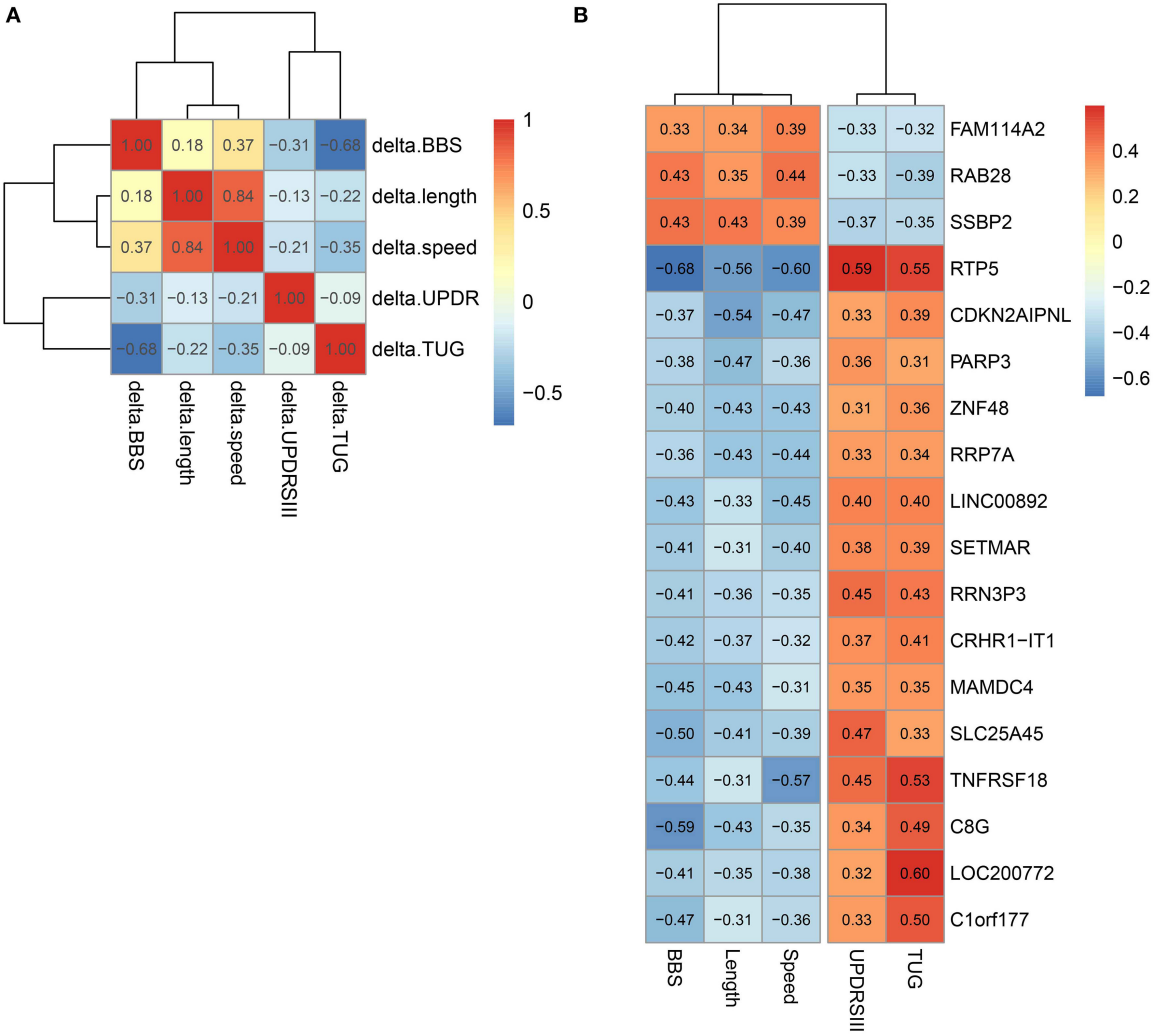
Correlation between different clinical measures and different genes. **(A)** Correlation between different clinical measures. **(B)** Genes correlated with all five clinical measures.

We studied the relationship between clinical measures and transcriptome of blood leukocytes by Pearson correlation coefficients. The Pearson correlation test was conducted between the changes of five clinical measures after exercise, and a highly significant correlation (threshold > 0.5) was identified between length and speed (both were measures for movement; Figure 3A). Moreover, TUG showed a highly significant correlation (*r* = 0.68) with BBS. Both measured the function of balance. We did not find a highly significant correlation between UPDRS and measures for movement and balance. Analysis of the BBS-correlated genes, which was measured for balance, showed that the most enriched signaling pathway was the T-cell receptor signaling pathway (Table 1). Interestingly, the T-cell receptor signaling pathway was also the enriched signaling pathways for length correlated genes (Table 1). For TUG speed, and UPDRSIII, very few correlated genes were identified (Table 1).

**Table 1:** Correlation between Kyoto Encyclopedia of Genes and Genomes (KEGG) pathways and clinical measures.

**KEGG ID**	**pBH**	**Odds ratio**	**Term**
**POSITIVE CORRELATION WITH BBS**			
565	0.0170	10.31	Ether lipid metabolism
4330	0.0179	7.72	Notch signaling pathway
3008	0.0429	4.38	Ribosome biogenesis in eukaryotes
**NEGATIVE CORRELATION WITH BBS**			
4660	0.0000	8.77	T cell receptor signaling pathway
5340	0.0000	15.28	Primary immunodeficiency
4640	0.0002	5.87	Hematopoietic cell lineage
5142	0.0112	3.64	Chagas disease (American trypanosomiasis)
5200	0.0112	2.32	Pathways in cancer
5322	0.0112	3.17	Systemic lupus erythematosus
4514	0.0247	2.79	Cell adhesion molecules (CAMs)
5020	0.0383	4.62	Prion diseases
5330	0.0383	4.35	Allograft rejection
**POSITIVE CORRELATION WITH LENGTH**			
4380	0.0021	3.53	Osteoclast differentiation
4662	0.0038	4.20	B-cell receptor signaling pathway
4910	0.0045	3.00	Insulin signaling pathway
5212	0.0045	4.05	Pancreatic cancer
4210	0.0053	3.53	Apoptosis
4370	0.0053	3.68	VEGF signaling pathway
4664	0.0053	3.52	Fc epsilon RI signaling pathway
4660	0.0053	3.05	T-cell receptor signaling pathway
5223	0.0053	4.20	Non-small cell lung cancer
4666	0.0053	3.22	Fc gamma R-mediated phagocytosis
5120	0.0055	3.69	Epithelial cell signaling in *Helicobacter pylori* infection
4722	0.0059	2.78	Neurotrophin signaling pathway
4620	0.0080	2.94	Toll-like receptor signaling pathway
5131	0.0081	3.64	Shigellosis
5142	0.0081	2.87	Chagas disease (American trypanosomiasis)
5214	0.0108	3.39	Glioma
5145	0.0161	2.43	Toxoplasmosis
4012	0.0166	2.79	ErbB signaling pathway
4650	0.0166	2.35	Natural killer cell mediated cytotoxicity
5220	0.0166	2.97	Chronic myeloid leukemia
4520	0.0166	2.97	Adherens junction
4330	0.0196	3.51	Notch signaling pathway
4150	0.0297	3.13	mTOR signaling pathway
4310	0.0297	2.10	Wnt signaling pathway
5160	0.0307	2.16	Hepatitis C
4720	0.0324	2.67	Long-term potentiation
5211	0.0324	2.67	Renal cell carcinoma
5140	0.0347	2.58	Leishmaniasis
4810	0.0347	1.84	Regulation of actin cytoskeleton
5221	0.0347	2.82	Acute myeloid leukemia
450	0.0364	5.10	Selenocompound metabolism
4360	0.0438	2.02	Axon guidance
4062	0.0478	1.78	Chemokine signaling pathway
4930	0.0478	2.78	Type II diabetes mellitus
520	0.0478	2.78	Amino sugar and nucleotide sugar metabolism
4670	0.0478	2.02	Leukocyte transendothelial migration
4140	0.0478	3.18	Regulation of autophagy
10	0.0478	2.43	Glycolysis/gluconeogenesis
**NEGATIVE CORRELATION WITH LENGTH**			
4660	0.0000	4.08	T-cell receptor signaling pathway
5340	0.0000	8.02	Primary immunodeficiency
100	0.0010	9.15	Steroid biosynthesis
4710	0.0134	5.80	Circadian rhythm—mammal
4514	0.0156	2.35	Cell adhesion molecules (CAMs)
4640	0.0229	2.55	Hematopoietic cell lineage
4612	0.0229	2.66	Antigen processing and presentation
3020	0.0229	4.11	RNA polymerase
4650	0.0252	2.11	Natural killer cell mediated cytotoxicity
514	0.0351	2.96	Other types of O-glycan biosynthesis
5020	0.0358	3.28	Prion diseases
5215	0.0363	2.23	Prostate cancer
604	0.0392	4.90	Glycosphingolipid biosynthesis—ganglio series
3008	0.0436	2.19	Ribosome biogenesis in eukaryotes
5332	0.0480	2.73	Graft-versus-host disease
**POSITIVE CORRELATION WITH SPEED**			
230	0.0332	4.22	Purine metabolism
1100	0.0480	1.95	Metabolic pathways
**NEGATIVE CORRELATION WITH SPEED**			
5322	0.0000	11.30	Systemic lupus erythematosus
5020	0.0005	16.24	Prion diseases
5150	0.0019	9.83	Staphylococcus aureus infection
4610	0.0034	7.70	Complement and coagulation cascades
4514	0.0260	3.84	Cell adhesion molecules (CAMs)
**POSITIVE CORRELATION WITH UPDRS III**			
534	0.0340	8.73	Glycosaminoglycan biosynthesis—heparan sulfate
3008	0.0340	4.53	Ribosome biogenesis in eukaryotes
340	0.0340	7.72	Histidine metabolism
3410	0.0364	6.69	Base excision repair
5200	0.0475	2.19	Pathways in cancer
310	0.0475	4.88	Lysine degradation
5222	0.0475	3.31	Small cell lung cancer
330	0.0480	3.92	Arginine and proline metabolism
**NEGATIVE CORRELATION WITH UPDRS III**			
4621	0.0429	6.16	NOD-like receptor signaling pathway
4380	0.0429	3.67	Osteoclast differentiation
4664	0.0429	4.44	Fc epsilon RI signaling pathway
4080	0.0429	2.61	Neuroactive ligand-receptor interaction
**POSITIVE CORRELATION WITH TUG**			
3040	0.0346	4.77	Spliceosome

In all genes, 18 genes that significantly correlated with all the five clinical measures were identified (Figure 3B). Among them, we noted that the correlation between the clinical indicators and RTP5 and TNFRSF18 were the strongest, and both of them were downregulated after exercise. For RTP5, we found that it had a highly significant correlation (threshold > 0.5) with all the five clinical parameters. But since it was a newly annotated gene, the function of RTP5 remained largely unknown. TNFRSF18, which encoded receptor, involved T-cell activation and interactions between activated T-lymphocytes and endothelial cells (37). The significant downregulation of TNFRSF18 (Table S4) was confirmed by qPCR in group 2.

### Genes for T-Cell Activation and Differentiation Were Upregulated in PD

**Figure 4:**
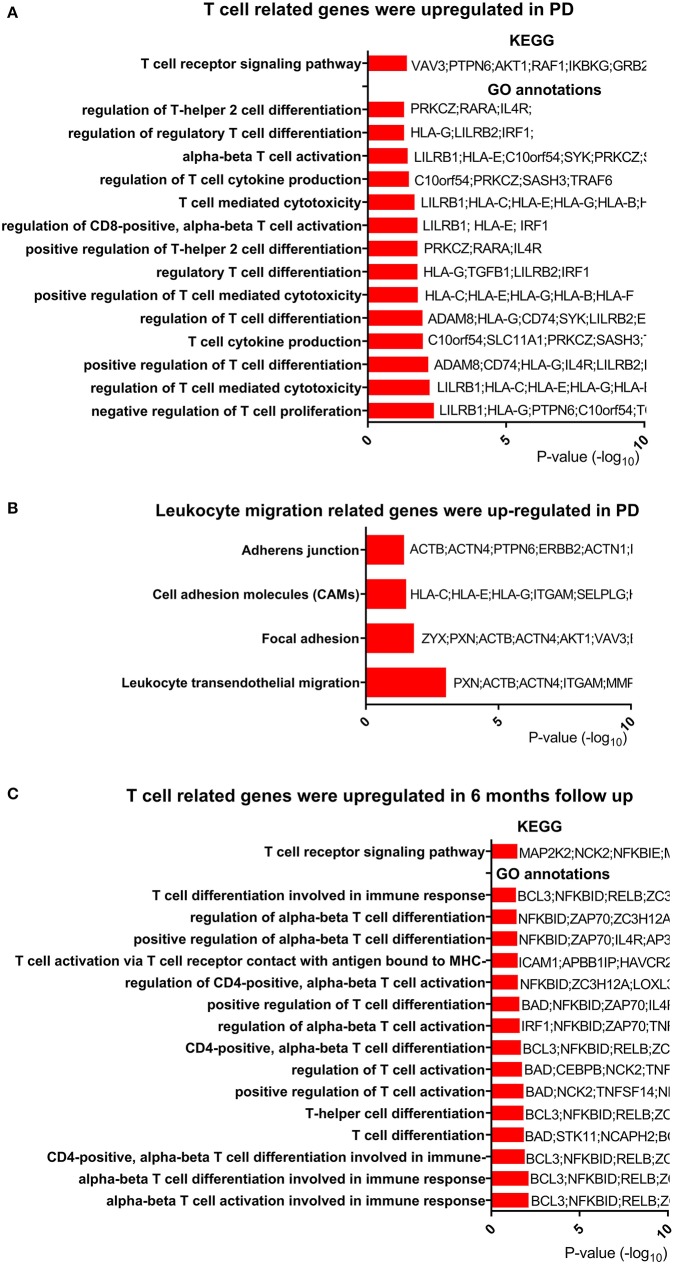
T-cell-related genes were regulated in PD compared with control. **(A)** T-cell-related genes were upregulated in Parkinson's disease (PD). Only T-cell-related terms are presented here. The completed data of Kyoto Encyclopedia of Genes and Genomes (KEGG) pathway analysis and enriched Gene Ontology (GO) annotations are available in Tables S7, S8. **(B)** Leukocyte migration-related genes were upregulated in PD. The completed data of KEGG are available in Table S7. **(C)** T-cell-related genes were upregulated after 6 months in PD. The completed data of KEGG and enriched GO annotations are available in Tables S9, S10.

Based on the change in T-cell-related pathways after exercise and its relationship with clinical measures, we hypothesized that T-cell-related functions, especially the T-cell receptor signaling pathway, were dysregulated in PD patients. To test this hypothesis, we reanalyzed publicly available microarray data (GSE99039) (22), which included peripheral blood from 205 PD patients and 233 control subjects. Genes (637 upregulated and 177 downregulated) were identified as DEGs in PD patients. Function enrichment analysis revealed that the T-cell receptor signaling pathway was among the most upregulated pathways in PD patients (Figure 4A, Table S7). We also noted that pathways for regulation of T-cell activation and differentiation were upregulated in PD patients (Figure 4A, Table S8). Among the DEGs, we found TNF receptor superfamily member 9 (TNFRSF9), which plays an essential role in T-cell activation, was upregulated. Both TNFRSF9 and TNFRSF18 belonged to TNF receptor superfamily. Interestingly, we found that genes for leukocyte transendothelial migration were upregulated in PD patients (Figure 4B).

To explore the change in T-cell-related function over time, we analyzed another microarray data from PPMI study downloaded on February 5th, 2019. Two hundred ninety-four PD patients with peripheral blood samples from baseline and 6 months after baseline were obtained from this microarray data. In the function enrichment, we found that T-cell receptor signaling pathway was upregulated after 6 months (Figure 4C, Table S9). In addition, T-cell activation and differentiation programs were also upregulated (Figure 4C, Table S10). In summary, these results showed that T cell was dysfunctional in PD patients and that leukocyte migration was activated in those patients.

## Discussion

Previous studies (2, 3) have shown that movement rehabilitation can improve movement and balance in patients with PD, which is consistent with our previous research. The present study also supports that patients with PD have different gene expression signatures before and after exercise. The gene expression changes suggest that exercise can modulate the abnormal immunity in patients with PD through T-cell-related function.

Although a consensus of the effectiveness of exercise in the management of PD has long been reached, the underlying pathomechanisms remain controversial. The immune system, especially the central immune system, has recently been recognized as a significant feature of PD pathophysiology. Chronic neuroinflammation, Lewy body inclusions, and loss of dopaminergic neurons in the substantia nigra parts compacta of the midbrain characterize PD pathology (38). Microglial activation and increased expression of inflammatory genes make up the *in vivo* evidence for neuroinflammation in PD patients. T-cell infiltration has been observed in affected brain areas in neurodegenerative diseases (39, 40), and the discovery of peripheral lymphocytes involving the progression of PD and hyperactivity in response to LPS stimulation (17, 41) shows that an inflammatory process is ongoing in the central nervous system and it may also be reflected in the peripheral circulation. For leukocyte microarray experiments, two studies have already been completed with more than 100 PD patients in each study (22, 42). Both studies focused on the molecular markers of PD, and they reported that functions related to metabolism, oxidation, and ubiquitination/proteasomal activity are dysregulated in PD patients. However, the studies did not pay much attention to the change in immune-related functions. Reanalysis of the first large microarray experiment highlighted modified neuroimmune signaling in blood cells from PD patients (43). Recently, DNA methylation alterations analysis of the second microarray experiment reveals that the most affected functions in PD by the methylation alterations are immune related, especially T-cell activation and T-cell receptor signaling pathway (44). Based on the dysfunction of the immune system in PD patients, modulating neuroinflammation has been proven effective in reducing T-cell infiltration and preventing the degeneration of dopaminergic neurons (24).

In this study, we tried to explore the mechanisms of exercise improving clinical symptoms of PD patients through RNA-Seq of circulating leukocyte collected before and after exercise. Previous studies showed exercise could induce a robust change in leukocyte function (45, 46). In this study, we found that patients with PD had different gene expression signatures before and after a 12 weeks exercise program. We identified some PD-related biological function genes that were regulated after exercise, such as ODC1 and TRAF3. ODC1 is thought to be associated with mitochondrial dysfunction (47), while TRAF3 plays an important role in the ubiquitin–proteasome system (34). Furthermore, after exercise both LGMN and AGAP2, which have been proved to regulate α-synuclein-related etiopathological effect, exhibited a pattern indicating amelioration of PD pathology. Besides the PD pathology-related pathways and biological function, the KEGG pathway and GO analysis highlighted that immune-related pathways and biological functions were regulated after exercise. The results support that exercise can modulate the abnormal gene expression patterns in leukocytes of PD patients.

DEG analysis also showed the TNFRSF18, a T-cell activation related gene was among the most downregulated genes after exercise and had the strongest correlation with clinical measures. Among those regulated pathways and biological processes, we found that the T-cell receptor signaling pathway was most involved before and after exercise, and it had a significant correlation with our clinical measures. In GSE99039 and PPMI microarray experiments, the T-cell receptor signaling pathway was upregulated in PD patients and further upregulated after 6 months, while T-cell receptor signaling pathway and T-cell-regulated genes were downregulated after exercise. It is suggested that exercise can ameliorate the aberrant of T-cell-regulated genes in PD patients. In this study, we also found that genes for T-cell transendothelial migration were upregulated in PD patients. Previous studies have proved that brain-infiltrated T cell plays a crucial role in the degeneration of dopaminergic neurons (24, 39). These results support the hypothesis that exercise can affect the degeneration of dopaminergic neurons by modulating T-cell activation and migration.

Another most affected pathway and biological process in our study was the cytokine–cytokine receptor interaction. In patients with PD, it has been demonstrated that their serum TNF level, an area that appears to be more intensely studied than other inflammatory factors (48), was elevated. In this study, we observed that TNF was upregulated after exercise in both messenger RNA and serum protein levels, which was consistent with findings from studies of exercise in normal participants whose gene expression levels of TNF were increased after exercise (49, 50). Although exercise upregulates TNF, we did not find a significant correlation between the TNF level and any of the clinical measures. IL-6 and IL-2R were elevated in the blood of PD patients and were significantly correlated with more severe symptoms (48). In this study, both IL-6 and IL-2R showed no significant change in serum after exercise, and they were not significantly correlated to clinical measures. These observations suggest that IL-6 and IL-2R may not be the key cytokines that control or mediate the effect of exercise in PD patients. More studies are required to identify key regulators that mediate the exercise effect.

In addition, epidemiological studies have suggested that persons with a history of moderate to vigorous exercise might have a reduction in PD risk (51). However, the research on the relationship between exercise and PD risk mainly focuses on neuroprotection. This study suggests that exercise plays a positive role in immune cells of PD patients, which may shed new light on the understanding of how exercise can reduce the risk of PD.

Although our RNA-Seq results were confirmed in the second group of PD patients, the results of this study might be vulnerable to bias. For example, the change in climate during the exercise could affect gene expression results. Moreover, we found that T-cell-related genes and functions were regulated after exercise, and they had a strong correlation with clinical measures. However, it remained unclear whether those changes directly improved the clinical indicators or whether they were just secondary responses to the improved clinical indicators. In addition, only 43 patients were enrolled in this study; larger-scale clinical researches with more measures would be more convincing to verify our findings.

## Conclusion

In conclusion, our study found that exercise rehabilitation improved movement and balance in PD patients and changed the genes expression profile in their peripheral leukocytes. Based on pathway analysis, exercise decreased genes associated with T-cell activation, suggesting that exercise ameliorates PD symptoms by reducing peripheral inflammation. Our findings suggest that attenuating T-cell activation by exercise may be useful to complement pharmacotherapy for the control of PD symptoms.

## Data Availability Statement

The datasets generated for this study can be found in Gene Expression Omnibus (GEO), GSE124676.

## Ethics Statement

This study was carried out in accordance with the recommendations of the Ethics Committee of Tongji Hospital with written informed consent from all subjects. All subjects gave written informed consent in accordance with the Declaration of Helsinki. The protocol was approved by the Ethics Committee of Tongji Hospital.

## Author Contributions

LJ and SL contributed conception and design of the study. FC supervised the project. JW and YS prepared the experimental setup. TZ, JW, WQ, WT, QG, CG, XC, and YHe collected the data. YHu and KZ performed the statistical analysis. LJ, UK, SL, YS, YHu, and KZ discussed the findings. YHu and KZ wrote the first draft of the manuscript. All authors contributed to manuscript revision and read and approved the submitted version.

### Conflict of Interest

The authors declare that the research was conducted in the absence of any commercial or financial relationships that could be construed as a potential conflict of interest.

## Supplementary Material

Figure S1 Flow diagram of the study.

Figure S2 Gene expression change trend for AGAP2, LGMN, and CEBPB.

Figure S3 The effect of exercise on inflammatory factors. **(A)** TNF-α level in blood plasma. **(B)** IL-2R level in blood plasma. **(C)** IL-6 level in blood plasma. Analyses were performed with paired *t*-tests. PD patients have higher levels of TNF-α after the training (*p* < 0.01). No significant difference is observed for IL2-R and IL-6. Red, increase after exercise; blue, decrease after exercise; black, no change after exercise.

Table S1 Primer sequences used to quantify gene expression by RT-qPCR.

Table S2 Clinical features of the study.

Table S3 Differentially expressed genes list after exercise.

Table S4 Validation of selected genes' expression in an independent cohort by RT-qPCR.

Table S5 KEGG analysis for differentially expressed genes after exercise.

Table S6 GO annotations for differentially expressed genes after exercise.

Table S7 KEGG analysis for differentially expressed genes between Parkinson's disease and control.

Table S8 GO annotations for differentially expressed genes between Parkinson's disease and control.

Table S9 KEGG analysis for differentially expressed genes between new-onset Parkinson's disease and 6 months follow-up.

Table S10 GO annotations for differentially expressed genes between new-onset Parkinson's disease and 6 months follow-up.
