# Supplementary figures and images for: Exercise Reverses Dysregulation of T-Cell-Related Function in Blood Leukocytes of Patients With Parkinson's Disease
Source: Front Neurol. 2020 Jan 28;10:1389. doi: 10.3389/fneur.2019.01389 (PMC6997272; doi:10.3389/fneur.2019.01389)

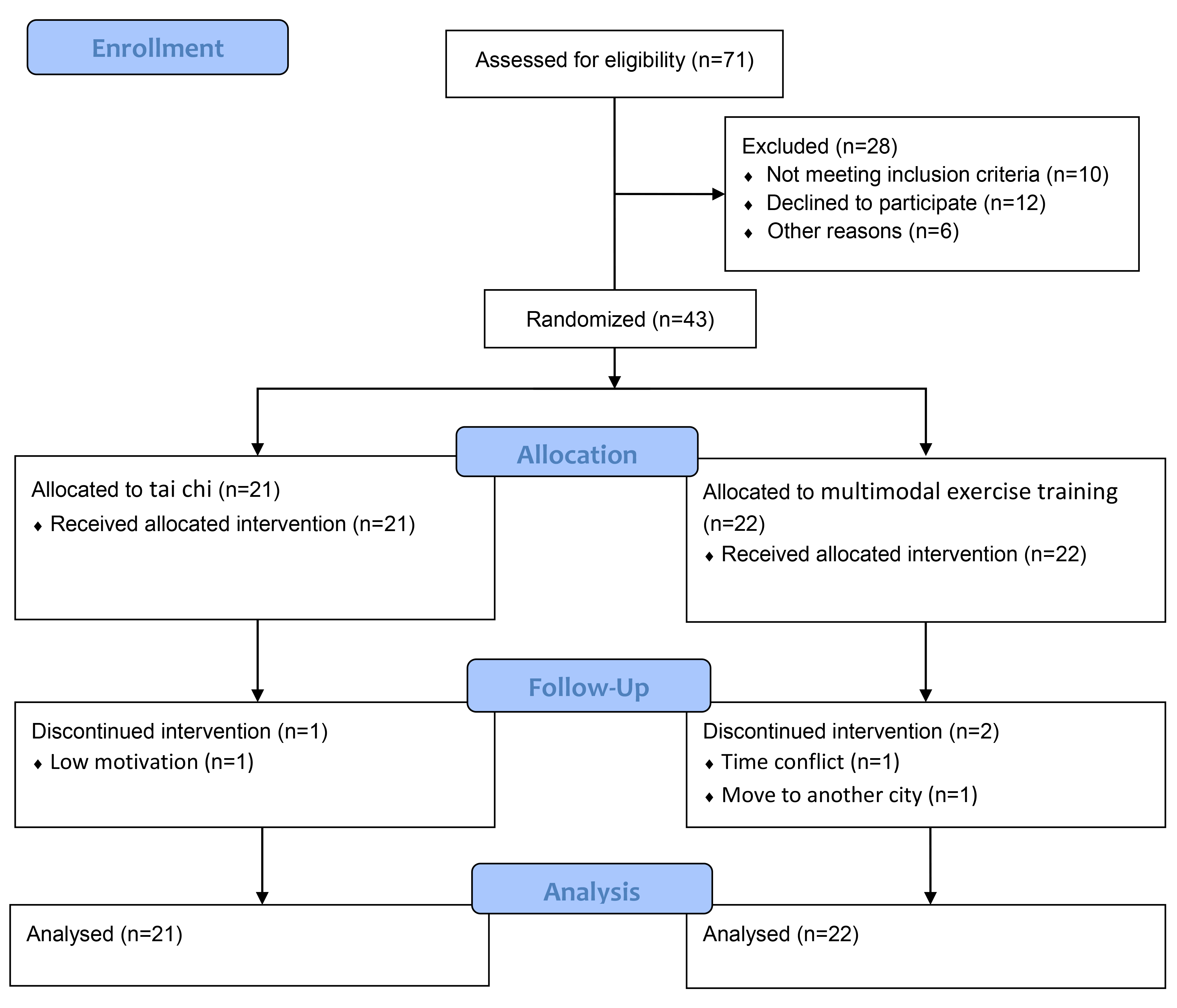

Supplement: Figure S1 — Flow diagram of the study. [file Image_1.JPEG]

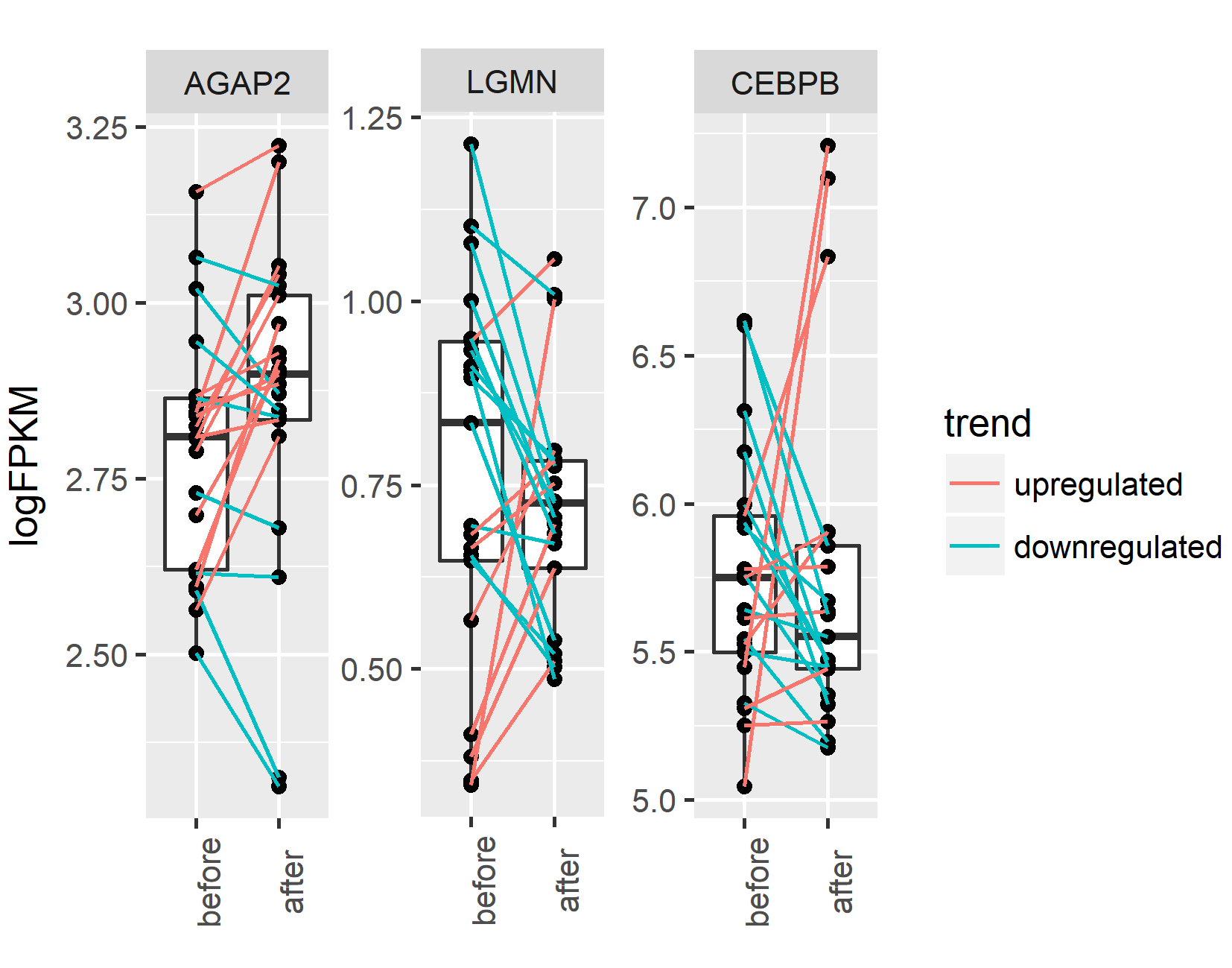

Supplement: Figure S2 — Gene expression change trend for AGAP2, LGMN, and CEBPB. [file Image_2.JPEG]

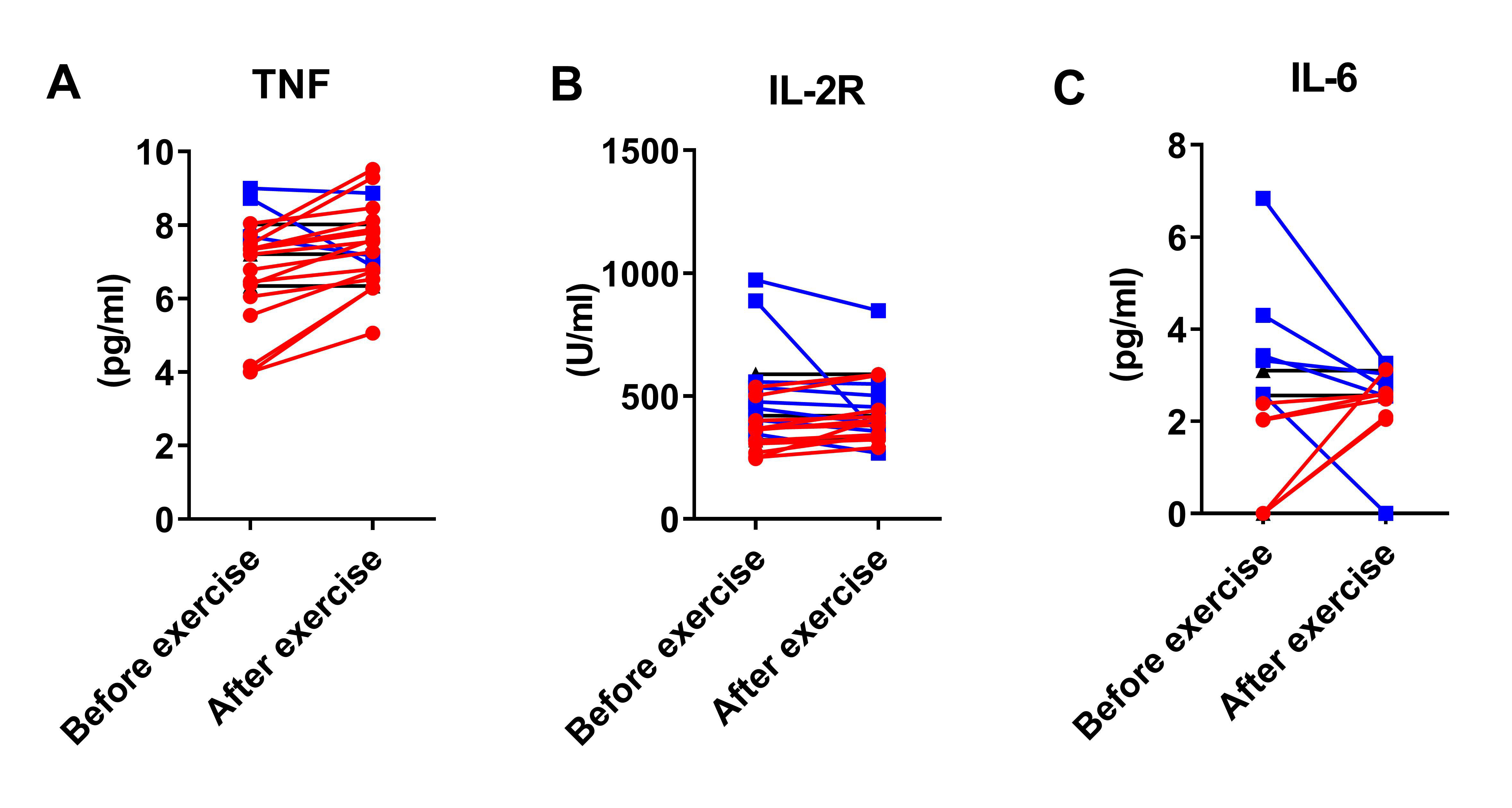

Supplement: Figure S3 — The effect of exercise on inflammatory factors. (A) TNF-α level in blood plasma. (B) IL-2R level in blood plasma. (C) IL-6 level in blood plasma. Analyses were performed with paired t-tests. PD patients have higher levels of TNF-α after the training (p < 0.01). No significant difference is observed for IL2-R and IL-6. Red, increase after exercise; blue, decrease after exercise; black, no change after exercise. [file Image_3.JPEG]
