# Supplementary material for: Exercise Reverses Dysregulation of T-Cell-Related Function in Blood Leukocytes of Patients With Parkinson's Disease
Source: Front Neurol. 2020 Jan 28;10:1389. doi: 10.3389/fneur.2019.01389 (PMC6997272; doi:10.3389/fneur.2019.01389)
Supplement: Table S1 — Primer sequences used to quantify gene expression by RT-qPCR. [file Table_1.DOC]

Supplemental files:

Table S1. Primer sequences used to quantify gene expression by RT-qPCR

| Gene | Primer (5'→3') |
| --- | --- |
| ODC1 | CTGCTTGATATTGGCGGTGG |
|  | ACGGAAAGTATTTGTCCAACGC |
| TRAF3 | TGTCGGAATGAAAGCAGAGGT |
|  | AGGACGCACACATGGAAGTT |
| OGFRL1 | AACTGGAAATGTTGCTCGGG |
|  | TATCCAAGCTCACCAAGGCT |
| CD3E | CAGAGGAAGCAAACCAGAAGATG |
|  | ATGACAATTGTGGCCACCGA |
| GRAP2 | GTGAAATGAATGCGGCCCTC |
|  | GCCTCAAAGTCATACAGCGC |
| FOS | TACTACCACTCACCCGCAGA |
|  | CGTGGGAATGAAGTTGGCAC |
| ZAP70 | CCTTCATCGAGCAGGGCAA |
|  | GGCGATCCTCCCACTTGTAG |
| TNFRSF18 | TGAATTCCACTGCGGAGACC |
|  | GCAGTCTGTCCAAGGTTTGC |
| GAPDH | ACAGCCTCAAGATCATCAGCA |
|  | TGGTCATGAGTCCTTCCACG |
