# Supplementary material for: Exercise Reverses Dysregulation of T-Cell-Related Function in Blood Leukocytes of Patients With Parkinson's Disease
Source: Front Neurol. 2020 Jan 28;10:1389. doi: 10.3389/fneur.2019.01389 (PMC6997272; doi:10.3389/fneur.2019.01389)
Supplement: Table S2 — Clinical features of the study. [file Table_2.DOC]

Supplemental files:

Table S2. Clinical features of the study cohorts

|  | **Tai chi (n=21)** | **MET (n=22)** | ***p*-value** |
| --- | --- | --- | --- |
| Sex (male/female), n | 14/7 | 14/8 | 0.835 |
| Body Mass Index (SD) | 23.67±2.82 | 24.30±3.15 | 0.495 |
| Hoehn and Yahr stage, n |  |  | 0.090 |
| 1 | 5 | 2 |  |
| 1.5 | 2 | 4 |  |
| 2 | 8 | 3 |  |
| 2.5 | 5 | 10 |  |
| 3 | 1 | 3 |  |
| Age, mean (SD), yrs | 65.71±11.85 | 67.68±10.47 | 0.567 |
| Duration of disease, mean (SD), yrs | 4.98±3.16 | 4.86±3.48 | 0.912 |
| MMSE, mean (SD) | 27.81±2.32 | 26.95±1.91 | 0.193 |
| Levodopa equivalent doses, mean (SD), mg/day | 360.20±226.61 | 384.80±249.42 | 0.737 |

MET, multimodal exercise training;

MMSE, Mini-Mental State Examination;

Levodopa equivalents doses were based on the following calculations: 100 mg of levodopa equals 133 mg of controlled-release levodopa, 100 mg of piribedil, 10 mg of selegiline-oral, 100 mg of amantadine, 1 mg of rasagiline, and 1 mg of pramipexole.
