# Supplementary material for: Exercise Reverses Dysregulation of T-Cell-Related Function in Blood Leukocytes of Patients With Parkinson's Disease
Source: Front Neurol. 2020 Jan 28;10:1389. doi: 10.3389/fneur.2019.01389 (PMC6997272; doi:10.3389/fneur.2019.01389)
Supplement: Table S4 — Validation of selected genes' expression in an independent cohort by RT-qPCR. [file Table_4.DOC]

Supplemental files:

Table S4. Validation of selected genes’ expression in an independent cohort by RT-qPCR.

| **Gene** | **RNA-seq** | | **RT-qPCR** | |
| --- | --- | --- | --- | --- |
|  | **FC** | ***p*-value** | **FC** | ***p-*value** |
| ODC1 | 0.76 | 0.000041 | 0.51 | 0.009336 |
| TRAF3 | 1.13 | 0.000393 | 1.82 | 0.006108 |
| OGFRL1 | 1.31 | 0.000547 | 1.79 | 0.005698 |
| CD3E | 0.59 | 0.000236 | 0.66 | 0.037668 |
| GRAP2 | 0.78 | 0.001388 | 0.69 | 0.006477 |
| FOS | 0.37 | 0.003032 | 0.56 | 0.021637 |
| ZAP70 | 0.67 | 0.003547 | 0.71 | 0.007309 |
| TNFRSF18 | 0.19 | 0.000912 | 0.25 | 0.003673 |

FC, Gene expression fold changes
